# A Serious Case against a Hospital

**Published:** 1888-10-06

**Authors:** 


### "A SERIOUS CASE AGAINST A HOSPITAL."

Under this heading there appeared in last week's numb *Lloyd's News* an account of an inquest on a child, who, was asserted, did not receive proper attention at the London Hospital, to which she was taken for treatment. Doubting the statement, we immediately made inquiries on the spot, and gathered the following true particulars: On September 27th, about a quarter past seven, a little girl was brought to the London Hospital so badly burnt that the medical officer who saw her immediately sent the porter with her at once to the ward. As the man entered he called out, "Nurse, it is a burn," and, as usual in urgent cases, two nurses promptly came forward and took charge of the child. She was accompanied by an excited party of friends, all Jews like herself. The wounds were dressed, brandy and milk were given, and the child comfortably settled in a cot next the fire. In a short time more friends arrived, and startled the nurses by announcing they had brought a cab to take the child home. The house surgeon was summoned, and he remonstrated with the friends and explained the dangerous condition of the patient. The father insisted that the child should die at home, and offered to move her there and then wrapped in a flimsy shawl and a coat. The house surgeon said they would remove the child at their own risk, but they must send for a sufficient number of blankets to wrap her in, or he would not let her leave the hospital. To the last moment the nurses and sister tried to persuade the father to leave the child, but directly the blankets arrived the child was packed in them and carried to the cab, and so taken home to die. This case ought to be a warning to coroners and newspaper editors not to be too ready to spread ex parte statements likely to injure a splendidly-managed hospital like the London.

